# Abiotic Stress Responses and Microbe-Mediated Mitigation in Plants: The Omics Strategies

**DOI:** 10.3389/fpls.2017.00172

**Published:** 2017-02-09

**Authors:** Kamlesh K. Meena, Ajay M. Sorty, Utkarsh M. Bitla, Khushboo Choudhary, Priyanka Gupta, Ashwani Pareek, Dhananjaya P. Singh, Ratna Prabha, Pramod K. Sahu, Vijai K. Gupta, Harikesh B. Singh, Kishor K. Krishanani, Paramjit S. Minhas

**Affiliations:** ^1^Department of Microbiology, School of Edaphic Stress Management, National Institute of Abiotic Stress Management, Indian Council of Agricultural ResearchBaramati, India; ^2^Stress Physiology and Molecular Biology Laboratory, School of Life Sciences, Jawaharlal Nehru UniversityNew Delhi, India; ^3^Department of Biotechnology, National Bureau of Agriculturally Important Microorganisms, Indian Council of Agricultural ResearchKushmaur, India; ^4^Molecular Glyco-Biotechnology Group, Discipline of Biochemistry, School of Natural Sciences, National University of Ireland GalwayGalway, Ireland; ^5^Department of Chemistry and Biotechnology, ERA Chair of Green Chemistry, School of Science, Tallinn University of TechnologyTallinn, Estonia; ^6^Department of Mycology and Plant Pathology, Institute of Agricultural Sciences, Banaras Hindu UniversityVaranasi, India

**Keywords:** abiotic stress, genomics, metabolomics, microbes, multi-omics, plant–microbe interactions

## Abstract

Abiotic stresses are the foremost limiting factors for agricultural productivity. Crop plants need to cope up adverse external pressure created by environmental and edaphic conditions with their intrinsic biological mechanisms, failing which their growth, development, and productivity suffer. Microorganisms, the most natural inhabitants of diverse environments exhibit enormous metabolic capabilities to mitigate abiotic stresses. Since microbial interactions with plants are an integral part of the living ecosystem, they are believed to be the natural partners that modulate local and systemic mechanisms in plants to offer defense under adverse external conditions. Plant-microbe interactions comprise complex mechanisms within the plant cellular system. Biochemical, molecular and physiological studies are paving the way in understanding the complex but integrated cellular processes. Under the continuous pressure of increasing climatic alterations, it now becomes more imperative to define and interpret plant-microbe relationships in terms of protection against abiotic stresses. At the same time, it also becomes essential to generate deeper insights into the stress-mitigating mechanisms in crop plants for their translation in higher productivity. Multi-omics approaches comprising genomics, transcriptomics, proteomics, metabolomics and phenomics integrate studies on the interaction of plants with microbes and their external environment and generate multi-layered information that can answer what is happening in real-time within the cells. Integration, analysis and decipherization of the big-data can lead to a massive outcome that has significant chance for implementation in the fields. This review summarizes abiotic stresses responses in plants in-terms of biochemical and molecular mechanisms followed by the microbe-mediated stress mitigation phenomenon. We describe the role of multi-omics approaches in generating multi-pronged information to provide a better understanding of plant–microbe interactions that modulate cellular mechanisms in plants under extreme external conditions and help to optimize abiotic stresses. Vigilant amalgamation of these high-throughput approaches supports a higher level of knowledge generation about root-level mechanisms involved in the alleviation of abiotic stresses in organisms.

## Introduction

Adverse climatic conditions creating abiotic stresses are among the principal limiting factors for decline in agricultural productivity (Padgham, 2009; Grayson, 2013). As per the FAO report (2007), only 3.5% of the global land area has left unaffected by any environmental constraint^1^. Dominant abiotic stresses comprise drought, low/high temperature, salinity and acidic conditions, light intensity, submergence, anaerobiosis and nutrient starvation (Wang et al., 2003; Chaves and Oliveira, 2004; Agarwal and Grover, 2006; Nakashima and Yamaguchi-Shinozaki, 2006; Hirel et al., 2007; Bailey-Serres and Voesenek, 2008). Water deficit (drought) has affected 64% of the global land area, flood (anoxia) 13% of the land area, salinity 6%, mineral deficiency 9%, acidic soils 15% and, cold 57% (Mittler, 2006; Cramer et al., 2011). Out of the world’s 5.2 billion ha of dryland agriculture, 3.6 billion ha is affected by the problems of erosion, soil degradation and salinity (Riadh et al., 2010). Ruan et al. (2010) estimated salt affected soils to impact upon 50% of total irrigated land in the world costing US$12 billion in terms of loss (Flowers et al., 2010). Similarly, global annual cost of land degradation by salinity in irrigated lands could be US$ 27.3 billion due to loss in crop production (Qadir et al., 2014). The detrimental effect of salinity on plant growth is well established. The area under ever-increasing salinization has almost reached 34 million irrigated hectares (FAO, 2012)^2^. Although any accurate estimation of agricultural loss (reduction of crop production and soil health) in terms of agro-ecological disturbances due to abiotic stresses could not be made, it is evident that such stresses affect large land areas and significantly impact qualitative and quantitative loss in crop production (Cramer et al., 2011).

Plants frequently cope up with the rapid fluctuations and adversity of environmental conditions because of their intrinsic metabolic capabilities (Simontacchi et al., 2015). Variations in the outside environment could put the plant metabolism out of homeostasis (Foyer and Noctor, 2005), and create necessity for the plant to harbor some advanced genetic and metabolic mechanisms within its cellular system (Apel and Hirt, 2004; Gill and Tuteja, 2010). Plants possess an array of protective mechanisms acquired during the course of evolution to combat adverse environmental situations (Yolcu et al., 2016). Such mechanisms cause metabolic re-programming in the cells (Heil and Bostock, 2002; Swarbrick et al., 2006; Shao et al., 2008; Bolton, 2009; Massad et al., 2012) to facilitate routine bio-physico-chemical processes irrespective of the external situations (Mickelbart et al., 2015). Many times plants get facilitated in reducing the burden of environmental stresses with the support of the microbiome they inhabit (Turner et al., 2013a; Ngumbi and Kloepper, 2014).

Microbial life is the most fundamental and live system on the earth. Being important living component of the soils, they naturally become integral part of the crop production system as soon as a seed comes into the soil to start its life cycle. Microorganisms are important inhabitants of seeds also, and proliferate as the seeds grow in the soils to form symbiotic associations at the surface or endophytic interactions inside the roots, stems or leaves. Plant microbiome provides fundamental support to the plants in acquiring nutrients, resisting against diseases and tolerating abiotic stresses (Turner et al., 2013a). Microbial intrinsic metabolic and genetic capabilities make them suitable organisms to combat extreme conditions of the environment (Sessitsch et al., 2012; Singh et al., 2014). Their interactions with the plants evoke various kinds of local and systemic responses that improve metabolic capability of the plants to fight against abiotic stresses (Nguyen et al., 2016). A testament to the important attributes of the microbial interactions with plants is significant number of accumulating pieces of evidence that suggest in-depth mechanisms based on plant–microbe interactions that offer modulation of cellular, biochemical and molecular mechanisms connected with stress tolerance (Bakker et al., 2012; Onaga and Wydra, 2016). Growing interest in uncultured microbes, especially from the rhizosphere of the crop plants, depleted and degraded soils, soils with disturbed fertility status and endophytic communities that potentially represent ‘obligate endophytes’ inhabiting plant tissues deciphered multi-phasic functions associated with the stress tolerance in microbial communities. The advent of next-generation sequencing (NGS) facilities supported gradually increasing metagenomic work and consequently led to the accumulation of greater amount of data for functional characterization of microbial communities in the soils (Bulgarelli et al., 2012).

Work on plant–microbe interactions at biochemical, physiological and molecular levels established that microbial associations largely direct plant responses toward stresses (Farrar et al., 2014). For dissecting deeper interaction mechanisms and connecting the changes at molecular levels with the tolerance responses against stresses, biological data based on the multi-omics approaches were generated (Kissoudis et al., 2014). The data generation and analysis was supported by the advancements in the high-end instrumentation and computational integration which helped to decipher individual signal molecules, proteins, genes and gene cascades to connect them with the gene networks/pathways for their function description. Technological developments also facilitated understanding of gene editing systems, RNAi-mediated gene silencing, mutant technology, proteomic analysis and metabolite profiling to reveal voluminous molecular information that helped in improving our understanding of microbe-mediated mitigation strategies of abiotic stresses in plants (Yin et al., 2014; Luan et al., 2015). Multi-omics approaches have emerged as a holistic and integrated analytical strategies for the dissection of one of the most complex and dynamic living system of microbial interactions with plants and modulating the consequences developed in the plants to help them overcome stresses. In this review, we aim at summarizing the implications of abiotic stresses and plant responses generated thereafter in terms of biochemical and molecular mechanisms followed by the microbe-mediated stress mitigation processes. We further describe the role of multi-omics approaches in establishing understanding of plant–microbe interactions that help plants optimize abiotic stresses.

## How Do Abiotic Stresses Affect Plants?

**FIGURE 1:**
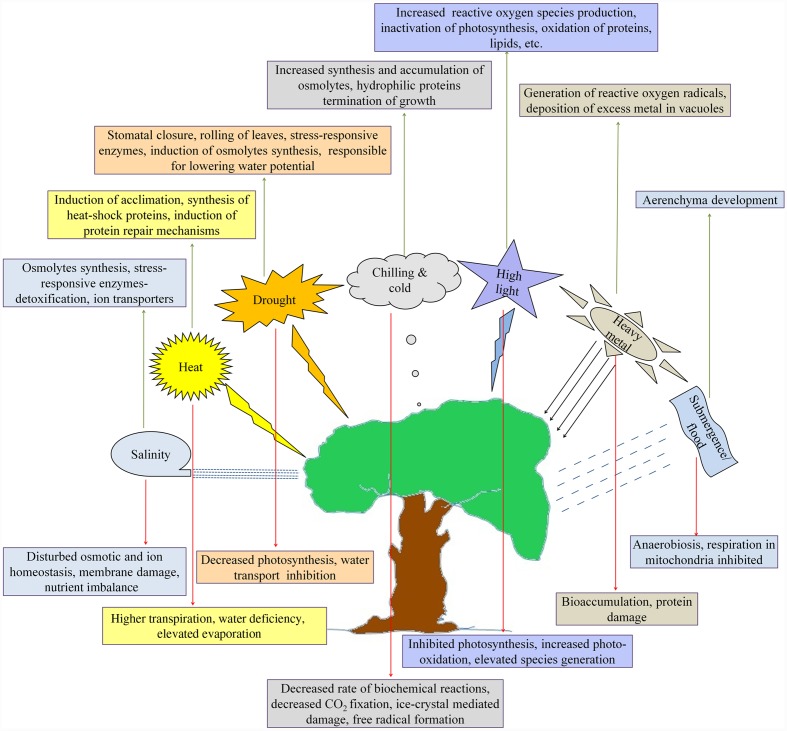
**Diverse abiotic stresses and the strategic defense mechanisms adopted by the plants.** Though the consequences of heat, drought, salinity and chilling are different, the biochemical responses seem more or less similar. High light intensity and heavy metal toxicity also generate similar impact but submergence/flood situation leads to degenerative responses in plants where aerenchyma are developed to cope with anaerobiosis. It is therefore, clear that adaptive strategies of plants against variety of abiotic stresses are analogous in nature. It may provide an important key for mounting strategic tolerance to combined abiotic stresses in crop plants.

Plants need light, water, carbon and mineral nutrients for their optimal growth, development and reproduction. Extreme conditions (below or above the optimal levels) limit plant growth and development. An unfavorable environment comprising extreme high or low of temperature, salinity and drought pose a complex set of stress conditions. Plants can sense and react to stresses in many ways that favor their sustenance (Crane et al., 2011; Ahmad et al., 2015; Jiang et al., 2016). They remember past exposure to abiotic stresses and even mechanisms to overcome them in such a way that responses to repeated stresses can be modified accordingly (Hilker et al., 2015). However, the underlying molecular mechanisms are primarily unknown. The most obvious effect of unfavorable conditions initially appear at the cellular levels after that, physiological symptoms are observable. Water stress adversely affects physiological status of plants including the photosynthetic capability (Xu and Zhou, 2006). Prolonged water stress decreases leaf water potential and stomatal opening, reduces leaf size, suppresses root growth, reduces seed number, size, and viability, delays flowering and fruiting and limits plant growth and productivity (Osakabe et al., 2014; Xu et al., 2016) (**Figure 1**). Therefore, plants have smartly evolved different mechanisms to minimize consumption of optimal water resources and manage their growth till they face adverse conditions (Osakabe et al., 2013). Exposure to low or high light intensities diminishes physiological process and adversely influences growth and development of plants. Excess light induces photooxidation that increases the production of highly reactive oxygen intermediates to manipulate biomolecules and enzymes (**Figure 1**). Under severe conditions, loss in plant productivity is observed (Li et al., 2009). Both freezing (cold) injury and/or an increase in temperature are major cause of crop loss (Koini et al., 2009; Pareek et al., 2010). Various edaphic factors like acidity, salinity, and alkalinity of soils (Bromham et al., 2013; Bui, 2013), pollutant contamination and anthropogenic perturbations (Emamverdian et al., 2015) severely affect plant development and adversely influence crop production (**Figure 1**). Different levels of acidic conditions badly influence soil nutrients and limit their ease of availability due to which plants become nutrient deficient and lose their normal physiological pattern of growth and development (Rorison, 1986). Early exposure to salinity leads to ion toxicity within the cell followed by disruption of osmotic balance when stress prolonged for longer duration. Combined effect of these ionic as well as osmotic shocks result into altered plant growth and development (Munns and Tester, 2008). Tolerance to salinity stress needs to maintain or quickly adjust both osmotic and ionic homeostasis within the cells. For combating salinity, plants usually try to avoid high saline environments by keeping sensitive plant tissues away from the zone of high salinity or by exuding ions from roots or compartmentalize ions away from the cytoplasm of physiologically active cells (Silva et al., 2010). Plants under extreme cold conditions survive either through avoiding super cooling of tissue water or through freezing tolerance. Certain species of plants have developed an ability to tolerate super-cooling or freezing temperatures by increasing their anti-freezing response within a short photoperiod, a process called cold acclimation (Thomashow, 2010).

After sensing the stress stimuli, plants exhibit an immediate and effective response to initiate a complex stress-specific signaling cascade (Chinnusamy et al., 2004; Andreasson and Ellis, 2010). Synthesis of phytohormone like abcisic acid, jasmonic acid, salicylic acid and ethylene (Spoel and Dong, 2008; Qin et al., 2011; Todaka et al., 2012), accumulation of phenolic acids and flavonoids (Singh et al., 2011; Tiwari et al., 2011), elaboration of various antioxidants and osmolytes and activation of transcription factors (TFs), are initiated along with the expression of stress-specific genes to mount appropriate defense system (Koussevitzky et al., 2008; Atkinson et al., 2013; Prasch and Sonnewald, 2013). Though many of the mechanisms related to stress tolerance in plants are known, our knowledge regarding ‘*on-field response*’ of the plants to simultaneous exposure to multiple stresses is still in quite an infancy.

The most crucial aspect in mitigating stress in plants is to understand fine level molecular machinery and its networks operative under stress conditions. This includes elaborative elucidation of abundance of metabolic pathways and their regulatory genes in the plant varieties. Identification of multigenic traits involved in stress responses, exploration of linked markers for such genes, and investigation of the probabilities to pool out important genes through breeding programs is the current focus of stress mitigation strategies. Other strategies that have put forward for the alleviation of abiotic stresses in plants include the use of various biomolecules of plant and microbial origin. These approaches are opening new gateways for scientists to dig out novel methods to alleviate the abiotic stresses in field grown plants.

## Physiological and Molecular Responses of Plants Against Stresses

Plants smartly sense, manage, maintain or escape changing environmental conditions (**Figure 1**). Their perception to environmental stimuli and responses to abiotic stresses involve an interactive metabolic crosstalk within diverse biosynthetic networks and pathways. Root architecture is thought to be more sensitive in sensing abiotic stimuli and reacting accordingly in the soils (Khan M.A. et al., 2016). It is a complex phenomenon that involves dynamic and real-time changes at genetic, transcriptomic, cellular, metabolic and physiological levels (Atkinson and Urwin, 2012). The foremost and direct impact of drought stress, frost, salinity and heat is creation of water deficient conditions within cells followed by a parallel development of biochemical, molecular and phenotypic responses against stresses (Cushman et al., 1990; Almoguera et al., 1995; Xu and Zhou, 2006). In the environment, the stresses experienced by the plants may be many, so as the complexity of their responses to multiple stresses in comparison to individual stress. The complexity lies in activating specific gene expression followed by metabolic programming in cells in response to individual stresses encountered. Tolerance, defense or susceptibility to stresses is a dynamic event involving multiple stages of plant’s development. Rather than imposing an additive effect on plants, abiotic stress responses may reduce or enhance susceptibility of plants toward biotic stresses caused due to pests or pathogens (Rizhsky et al., 2004). This becomes more important when we take into account agricultural crops because, in many agricultural systems, most crops grow in suboptimal environmental conditions that are limiting to the genetic potential of the plants for growth and development (Bray et al., 2000). Defense, repair, acclimation and adaptation are the major components of resistance responses toward stresses.

Plants are vulnerable to water stress. Environmental changes like rewatering or cycled water conditions are created most frequently in the globally changing climatic conditions (Xu et al., 2010). Under severe water deficit conditions, peroxidation may be induced leading to negative impact on antioxidant metabolism (Bian and Jiang, 2009; Xu et al., 2014). Rewatering further decreases the level of peroxidation and restores growth and development of new plant parts and stomatal opening. In roots, both drought and rewatering lead to high accumulation of H_2_O_2_ (Bian and Jiang, 2009). Drought responses vary from plant to plant in terms of the activity of superoxide dismutase (SOD) enzyme that plays a central role in antioxidant metabolism (Xu Z. et al., 2015). In bluegrass, SOD activity remains unaffected by drought conditions and gene expression of FeSOD and Cu/ZnSOD is down-regulated. In Alfalfa nodules, FeSOD and CU/ZnSOD are up-regulated by moderate drought, implicating that responses differ from species and tissues (Foyer and Noctor, 2005; Naya et al., 2007). An elevated level of salts present in the soil is detrimental to the plant cells, and different cells in a tissue respond differently to the stresses caused due to salinity (Voesenek and Pierik, 2008). Stressed cells irrespective of their location, whether at the root surface or within internal tissues, influence their neighbors and cause a change in their gene expression pattern over the stress duration (Dinneny et al., 2008). A drastic decrease in the osmotic potential of the soil occurs due to the elevated salt levels, the ultimate result of which is ion toxicity coupled with water stress in the plants. This situation can affect the vitality of the plants by suppressing seed germination and growth of the seedlings, hamper senescence of the plants and finally cause death (McCue and Hanson, 1990). The role of Salt Overly Sensitive (SOS) stress signaling pathway consisting of three majorly involved proteins SOS1, SOS2, and SOS3 is well demonstrated (Hasegawa et al., 2000). Salinity conditions cause decrease in the levels of aromatic amino acids like cysteine, arginine and methionine. Proline accumulation within the cells is a well-known alleviation strategy from salinity stress (Matysik et al., 2002). Similarly, generation of nitric oxide (NO), activation of antioxidant enzymes and compounds, modulation of hormones, accumulation of glycine betaine and polyols are some other changes within plants due to salinity stress (Gupta and Huang, 2014). This principally happens due to unavailability of water and mutilation in the nutrient availability caused due to high salt concentrations that create much damage to plant tissues and ultimately affect productivity.

Due to continued rise in global temperature, heat stress is becoming an important agricultural problem as it badly affects crop production. Rising temperature has an adverse impact on morpho-anatomical, physiological, biochemical and genetic changes in plants. A thorough understanding of physiological responses of plants to heat and mechanisms of tolerance could lead to strategic development of better approaches for crop production management (Wahid et al., 2007). Heat affects plants at different developmental levels, and high temperature causes reduced seed germination, loss in photosynthesis and respiration and decrease in membrane permeability (Xu et al., 2014). Alterations in the level of phytohormones, primary and secondary metabolites, enhancement in the expression of heat shock and related proteins and production of reactive oxygen species (ROS) are some prominent responses of plants against heat stress (Iba, 2002) (**Figure 1**). Mitigation strategies in plants against heat stress involve activation of mechanisms that support maintenance of membrane stability and induction of mitogen-activated protein kinase (MAPK) and calcium-dependent protein kinase (CDPK) cascades (Wang and Li, 2006). Besides, scavenging of ROS, accumulation of antioxidant metabolites and compatible solutes, chaperone signaling and transcriptional modulation are certain parallel activities that help cells to sustain heat stress (Wahid et al., 2007).

Multiple stress conditions impose more beneficial impacts on plants compared to that posed in presence of individual stress alone. Combination of stresses ultimately reduce the detrimental effect of each other thereby, increasing the probability of better survival of plants. Iyer et al. (2013) demonstrated that the cumulative impact of drought and accumulation of ozone (O_3_) in plants resulted in better tolerance. The combined affect was attributed to decreased values of stomatal conductance. Elevated concentration of reduced glutathione and ascorbic acid effectively scavenge ROS, thereby causing a considerable drop in the total ROS content. However, it is a difficult task to infer response pattern of a plant against any single stress, particularly when it is growing in the field from the cumulative impact of environmental stresses. Multiple stresses occur simultaneously in field conditions and so, multifaceted mechanisms exist in the plants to cope-up with rapidly fluctuating adverse situations. Although much efforts have been made to assess plant responses toward single stress conditions (Rizhsky et al., 2002, 2004; Mittler, 2006; Mittler and Blumwald, 2010; Alameda et al., 2012; Atkinson and Urwin, 2012; Kasurinen et al., 2012; Srivastava et al., 2012; Perez-Lopez et al., 2013; Rivero et al., 2013), attempts to assess the impact of combined stress conditions on crop plants under simulated laboratory trials are limited. This particularly limits our knowledge and understanding of plant responses to combined stresses and prediction of cumulative stress tolerance mechanisms in laboratory or field conditions.

Phytohormones are crucial for the plant growth and development but they critically play role in the abiotic stress tolerance (Wani et al., 2016). Gene expression profiling revealed that prioritization of signals done by protein switches like kinases, TFs and G-proteins are mostly regulated by hormones (Depuyd and Hardtke, 2011; Yao et al., 2011). Plants typically channel their physiological resources toward adapting to abiotic stress which makes them more susceptible to biotic stresses like herbivory and disease attack (Zabala et al., 2009; Hey et al., 2010). ABA-dependent abiotic stress response pathways are predominant. Other defense pathways rooted through salicylic acid, jasmonic acid or ethylene also trigger plants for abiotic stress response. For example, triggering ROS production to minimize loss during abiotic stress may prevent plants from biotrophic pathogen attack, but it makes plants more prone for necrotrophic pathogens. The other hormone, JA is effective for defense responses to necrotrophic pathogens and associated to ISR by beneficial microbes (Matilla et al., 2010). Study of omics may help in understanding these complex plant–microbe interactions and harvesting associated and linked understanding.

## Microbe-Mediated Mitigation of Abiotic Stresses

Microbial interactions with crop plants are key to the adaptation and survival of both the partners in any abiotic environment. Induced Systemic Tolerance (IST) is the term being used for microbe-mediated induction of abiotic stress responses. The role of microorganisms to alleviate abiotic stresses in plants has been the area of great concern in past few decades (de Zelicourt et al., 2013; Nadeem et al., 2014; Souza et al., 2015). Microbes with their potential intrinsic metabolic and genetic capabilities, contribute to alleviate abiotic stresses in the plants (Gopalakrishnan et al., 2015). The role of several rhizospheric occupants belonging to the genera *Pseudomonas* (Grichko and Glick, 2001; Ali et al., 2009; Sorty et al., 2016), *Azotobacter* (Sahoo et al., 2014a,b), *Azospirillum* (Creus et al., 2004; Omar et al., 2009), *Rhizobium* (Alami et al., 2000; Remans et al., 2008; Sorty et al., 2016), *Pantoea* (Amellal et al., 1998; Egamberdiyeva and Höflich, 2003; Sorty et al., 2016), *Bacillus* (Ashraf et al., 2004; Marulanda et al., 2007; Tiwari et al., 2011; Vardharajula et al., 2011; Sorty et al., 2016), *Enterobacter* (Grichko and Glick, 2001; Nadeem et al., 2007; Sorty et al., 2016), *Bradyrhizobium* (Fugyeuredi et al., 1999; Swaine et al., 2007; Panlada et al., 2013), *Methylobacterium* (Madhaiyan et al., 2007; Meena et al., 2012), *Burkholderia* (Barka et al., 2006; Oliveira et al., 2009), *Trichoderma* (Ahmad et al., 2015) and cyanobacteria (Singh et al., 2011) in plant growth promotion and mitigation of multiple kinds of abiotic stresses has been documented. Recently, Pandey et al. (2016) have demonstrated the role of *Trichoderma harzianum* on stress mitigation in rice genotypes due to upregulation of aquaporin, dehydrin and malonialdehyde genes along with various other physiological parameters. Rhizobacteria-induced drought endurance and resilience (RIDER) that includes changes in the levels of phytohormones, defense-related proteins and enzymes, antioxidants and epoxypolysaccharide have been observed for microbe-mediated plant responses. Such strategies make plants tougher toward abiotic stresses (Kaushal and Wani, 2016). The selection, screening and application of stress-tolerant microorganisms, therefore, could be viable options to help overcome productivity limitations of crop plants in stress-prone areas. Enhanced oil content in NaCl affected Indian mustard (*Brassica juncea*) was reported by *Trichoderma harzianum* application which improved the uptake of essential nutrients, enhanced accumulation of antioxidants and osmolytes and decreased Na^+^ uptake (Ahmad et al., 2015). Parallel to such reports, up-regulation of monodehydroascorbate reductase in *Trichoderma* treated plants was demonstrated. It was also confirmed by mutant studies that *Trichoderma* ameliorates salinity stress by producing ACC-deaminase (Brotman et al., 2013). In barley and oats, *Pseudomonas* sp. and *Acinetobacter* sp. were reported to enhance production of IAA and ACC-deaminase in salt affected soil (Chang et al., 2014). Palaniyandi et al. (2014) reported alleviation of salt stress and growth promotion by *Streptomyces* sp. strain PGPA39 in ‘Micro-Tom’ tomato plants. *Burkholderia phytofirmans* strain PsJN mitigates drought stress in maize (Naveed et al., 2014b), wheat (Naveed et al., 2014a) and salt stress in *Arabidopsis* (Pinedo et al., 2015).

The rhizosphere comprises the fraction of soil in vicinity of the plant roots. It constitutes a soil microenvironment in the proximity of root region where the average count of microorganisms is very high than rest of the bulk soil. It is, therefore, obvious that plant roots with a diversity of their nutrient, mineral and metabolite composition, could be a major factor responsible for attracting microorganisms to accumulate and associate alongside. The secretion of root exudates by plants is a vital factor for microbial colonization within the rhizosphere. Chemotactic movement of microorganisms toward the root exudates plays the role of dragging force for the microbial communities to colonize on the roots. While utilizing the rhizosphere-microenvironment around plant roots, the PGPRs may act as biofertilizers, phytostimulators or biocontrol agents depending upon their inherent capabilities, mode of interaction and competitive survival conditions. Growth promoting bacteria stimulate plant growth by employing several broadly categorized direct and indirect mechanisms (Braud et al., 2009; Hayat et al., 2010). Direct mechanisms include synthesis of bacterial compounds which facilitate uptake of essential nutrients and micronutrients from the soil along with the production of plant growth regulators, e.g., iron and zinc sequestration, siderophore production, phosphorus and potassium solubilisation, plant hormone production, and atmospheric nitrogen fixation. On the other hand, indirect mechanisms involve antagonistic activity toward plant pathogenic organisms, production of HCN and antifungal compounds and tolerance against abiotic stresses. Besides this, the bacteria can induce systemic resistance in plants by their metabolites acting as extracellular signals, which subsequently trigger a series of internal processes. Eventually, the translocated signal is perceived by the distant plant cells triggering the activation of the defense mechanism. Besides bacteria, fungi particularly the mycorrhiza are also important plant growth promoters. These are principally divided into mycorrhizal fungi and vesicular-arbuscular mycorrhizal (VAM) fungi. These fungi remain associated with the host plant externally (ectomycorrhizae) or they may form endosymbiotic associations (VAM). These fungi form extensive networking of very fine hyphae, thus increasing overall nutrient uptake by the roots. The root fungal endophyte *Piriformospora indica* induces salt tolerance in barley (Baltruschat et al., 2008) and drought tolerance in Chinese cabbage (Sun et al., 2010) by increasing the levels of antioxidants and improving many other aspects (Franken, 2012). The potential of microbial interactions with the plants have, therefore, multipronged role. At one end, microbes induce local or systemic stress alleviation response mechanisms in plants to sustain under abiotic stress conditions while at the other end, they help plants to maintain their growth and development through fixation, mobilization and/or production of nutrients, hormones and organic phytostimulant compounds. Such multifaceted action of microorganisms or their communities makes them strong, viable and vital options for abiotic stress mitigation strategies in crop plants.

Several mechanisms highlighting the role of microbes in abiotic stress alleviation have been proposed. Soil-inhabiting microbes belonging to genera *Achromobacter, Azospirillum, Variovorax, Bacillus, Enterobacter, Azotobacter, Aeromonas, Klebsiella* and *Pseudomonas* have been shown to enhance plant growth even under unfavorable environmental conditions (Pishchik et al., 2002; Hamdia et al., 2004; Mayak et al., 2004; Arkhipova et al., 2007; Barriuso et al., 2008a,b; Dardanelli et al., 2008; Belimov et al., 2009; Ortiz et al., 2015; Kaushal and Wani, 2016; Sorty et al., 2016). Literature relating to the involvement of microbes for the alleviation of abiotic stressors signifies the importance of microbes in this area (**Table 1**). All such soil bacteria that are capable of inducing plant growth under variety of physicochemical and environmental conditions are classified cumulatively as plant growth promoters (PGP). There exists different mechanisms by which microbes induce plant growth. The plant-growth regulating molecules predominantly, indole acetic acid (IAA) are synthesized in shoot and accumulated in the actively growing regions of roots. The IAA and other auxins have growth-stimulating effect in terms of cell elongation resulting in root growth initiation. Moreover, these molecules also promote the development of lateral roots. Higher concentrations of auxins, on the other hand, are known to have a negative impact on root growth (Jackson, 1991; Sorty et al., 2016). A similar situation can also happen due to increased synthesis of ethylene (Jackson, 1991). The rhizosphere colonizing bacteria were reported to perform in a similar manner, and produce phytohormones to enhance plant growth (Bowen and Rovira, 1991; Timmusk and Wagner, 1999; German et al., 2000; Belimov et al., 2007). Evidences from recent agricultural practices witness that the PGPRs not just help in mitigation of environmental stresses, but also improve yield of diverse crop plants including rice, maize, barley and soybean (Tapias et al., 2012; Sharma et al., 2013; Sen and Chandrasekhar, 2014; Suarez et al., 2015). A mechanism of salt tolerance imposed by *Pseudomonas* sp. PMDzncd2003 on rice germination under salinity stress is demonstrated. Better root colonizing capability of *Pseudomonas* sp. along with its ability to produce exopolysaccharides (EPS) leads to enhanced tolerance toward salinity (Sen and Chandrasekhar, 2014). Similarly, Khan A. et al. (2016) have shown that inoculation of *Bacillus pumilus* improved rice growth in response to salinity and high boron stresses. A possible mechanism was suggested, that higher expression of antioxidant enzyme machinery in the presence of bacterial inoculant may lead to cell protection in stress conditions. More efforts are now needed to dissect molecular mechanisms involved in the communication between plant and bacterial colonizers.

**Table 1:** Microbe-mediated abiotic stress tolerance in plants.

Abiotic stress	Microbe inoculation	Plant	Tolerance strategy	Reference
Salt	*Bacillus subtilis* GB03	*Arabidopsis thaliana*	Tissue-specific regulation of sodium transporter *HKT1*	Zhang et al., 2008
Salt	*Pseudomonas simiae*	*Glycine max*	4-nitroguaiacol and quinoline promote soybean seed germination	Vaishnav et al., 2016
Salt	*Pseudomonas syringae* DC3000, *Bacillus* sp. strain L81, *Arthrobacter oxidans*	*Arabidopsis thaliana*	SA-dependent pathway	Barriuso et al., 2008b
Salt	Root-associated plant growth-promoting rhizobacteria (PGPR)	*Oryza sativa*	Expression of salt stress-related *RAB18* plant gene	Jha et al., 2014
Salt	Cyanobacteria and cyanobacterial extracts	*Oryza sativa. Triticum aestivum. Zea mays, Gossypium hirsutum*	Phytohormones as elicitor molecule	Singh, 2014
Salt	*Pseudomonas koreensis* strain AK-1	*Glycine max* L. Merrill	Reduction in Na^+^ level and increase in K^+^ level	Kasotia et al., 2015
Osmotic stress	*Bacillus megaterium*	*Zea mays*	High hydraulic conductance, increased root expression of two ZmPIP isoforms	Marulanda et al., 2010
Osmotic stress	*Glomus intraradices* BEG 123	*Phaseolus vulgaris*	High osmotic root hydraulic conductance due to increased active solute transport through roots	Aroca et al., 2007
Salt	*Glomus etunicatum*	*Glycine max*	Increased root but decreased shoot proline concentrations	Sharifi et al., 2007
Salt	*Burkholderia, Arthrobacter* and *Bacillus*	*Vitis vinifera. Capsicum annuum*	Increased accumulation of proline	Barka et al., 2006
Drought	*Rhizobium tropici* and *Paenibacillus polymyxa* (Co-inoculation)	*Phaseolus vulgaris*	Upregulation of genes involved in stress tolerance	Figueiredo et al., 2008
Salt	*Glomus fasciculatum*	*Phragmites australis*	Accumulation of carbohydrates	Al-Garni, 2006
Salt	*Glomus intraradices*	*Glycine max*	Accumulation of carbohydrates	Porcel and Ruiz-Lozano, 2004
Salinity	*Azospirillum brasilense* and *Pantoea dispersa* (Co-inoculation)	*Capsicum annuum*	High stomatal conductance and photosynthesis	del Amor and Cuadra-Crespo (2012)
Salinity	*Glomus intraradices* BAFC 3108	*Lotus glaber*	Decreased root and shoot Na^+^ accumulation and enhanced root K^+^ concentrations	Sannazzaro et al., 2006
Salinity	*Glomus clarum Glomus etunicatum*	*Vigna radiata. Capsicum annuum. Triticum aestivum*	Decreased Na^+^ in root and shoot and incesaed concentration of K^+^ in root	Rabie, 2005; Daei et al., 2009; Kaya et al., 2009
Salinity	*Bacillus subtilis*	*Arabidopsis*	Decreased root transcriptional expression of a high-affinity K^+^ transporter (*AtHKT1*) decreasing root Na^+^ import	Zhang et al., 2008
Salinity	*Glomus intraradices* BEG121	*Lactuca sativa*	Reduced concentration of ABA	Aroca et al. (2008)
Salinity	*Pseudomonas putida* Rs-198	*Gossypium hirsutum*	Prevented salinity-induced ABA accumulation in seedlings	Yao et al., 2010
Salinity	*Azospirillum brasilense* strain Cd	*Phaseolus vulgaris*	Stimulation of persistent exudation of flavonoids	Dardanelli et al., 2008
Salinity	*Bacillus subtilis*	*Lactuca sativa*	Root-to-shoot cytokinin signalling and stimulation of shoot biomass	Arkhipova et al., 2007
Drought	*Burkholderia phytofirmans Enterobacter* sp. FD17	*Zea mays*	Increased photosynthesis, root and shoot biomass under drought conditions	Naveed et al., 2014b
Drought	*Bacillus thuringiensis* AZP2	*Triticum aestivum*	Production of volatile organic compounds	Timmusk et al., 2014
Drought	*Pseudomonas chlororaphis* O6	*Arabidopsis thaliana*	Production of 2R,3R butanediol- a volatile compound	Cho et al., 2008
Drought	*Pseudomonas putida* strain GAP-P45	*Helianthus annuus*	Epoxypolysaccharide production	Sandhya et al., 2009
Drought	*Bacillus licheformis* strain K11	*Capsicum annum*	Stress related genes and proteins	Lim and Kim, 2013
Drought	*Bacillus cereus* AR156, *B. subtilis* SM21 and Serratia sp. XY21	*Cucumis sativa*	Production of monodehydro ascorbate, proline, and antioxidant enzyme, expression of genes	Wang et al., 2012
Heat	*Bacillus amyloliquefaciens, Azospirillum brasilence*	*Triticum aestivum*	Reduced regeneration of reactive oxygen species, preactivation of heat shock transcription factors, changes in metabolome	El-Daim et al., 2014
Heat and drought	*Curvularia proturberata* isolate Cp4666D	*Dichanthelium lanuginosum, Solanum lycopersicum*	Colonization of roots	de Zelicourt et al., 2013
Arsenic toxicity	*Staphylococcus arlettae*	*Brassica juncea*	Increased soil dehydrogenase, phosphatase and available phosphorus	Srivastava et al., 2013
Pb/Zn toxicity	*Phyllobacterium myrsinacearum*	*Sedum plumbizincicola*	Resistance to 350mg/L Cd, 1000 mg/L Zn, 1200 mg/L Pb	Ma et al., 2013
Zn toxicity	*Pseudomonas aeruginosa*	*Triticum aestivum*	Improved biomass, N and P uptake and total soluble protein	Islam et al., 2014
Zn toxicity	*Enterobacter intermedius* MH8b	*Sinapis alba*	ACC deaminase, IAA, hydrocyanic acid, P solubilization	Plociniczak et al., 2013
Cd, AS, Cu, Pb and Zn toxicity	*Pseudomonas koreensis* AGB-1	*Miscanthus sinensis*	ACC deaminase, IAA production	Babu et al., 2015
Zn toxicity	*Pseudomonas brassicacearum, Rhizobium leguminosarum*	*Brassica juncea*	Metal-chellating molecules	Adediran et al., 2016
Hg toxicity	*Photobacterium* spp.	*Phragmites australis*	IAA, mercury reductase activity	Mathew et al., 2015


## Multi-Omics Approaches to Address Alleviation of Abiotic Stress

**FIGURE 2:**
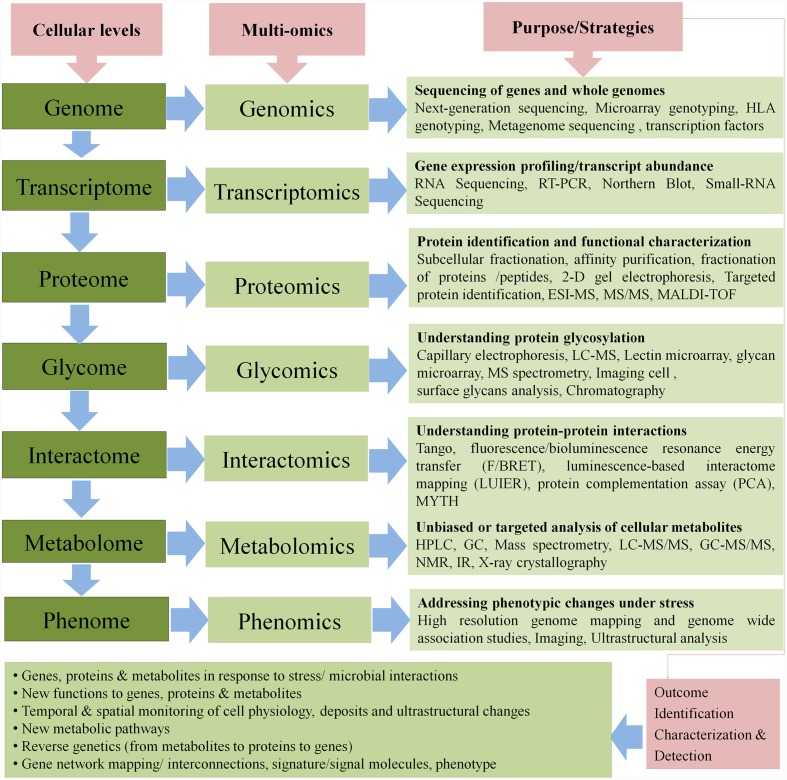
**Cellular level components, multi-omics approaches to address different levels and the strategies that help identify the outcome of the impact of abiotic stresses or impact of microbial-interactions**.

The ecology of plant–microbe interaction is very complicated and interwoven system. It is important to understand the fine-tuning and integration of diverse signals generated by microbial interactions in the plants for advantage in crop improvement. A plant has to combat multiple biotic and abiotic stresses in the environment. Multiple stress factors produce complex defense signals in plants and therefore, the result of plant–microbe interaction can be decided by prioritization of physiological pathways in plants (Schenk et al., 2012). Interaction of microbes with plant roots evoke multipronged responses in local and/or in distal plant parts at physiological, biochemical and molecular level. Such responses at all levels have their interconnections with the stress; many are parallel to stress responses while others are adverse. For dissecting the mechanisms, multi-omics approaches can be applied to address the challenging task of deciphering changes in plants at genetic, proteomic or metabolomic level (**Figure 2**). Entwined with the advances in bioinformatics, the data-driven science of multi-omics has improved our knowledge in understanding the microbial community composition and their functional behavior in complex environments like rhizosphere, where inter-connections among microbial communities direct plant responses toward stresses. Recently, meta-omics approaches including metagenomics, metatranscriptomics and metaproteomics have emerged as promising tools to address microbial communities and functions within a given environment at a deeper level (de Castro et al., 2013).

## Genomics

Abiotic stress alleviation by altering crop genetics is of paramount importance and is a challenging issue that requires extensive breeding programs (Grainger and Rajcan, 2013). Low heritability and environmental variations make such breeding programs even more challenging (Manavalan et al., 2009). Strategic marker-assisted breeding is efficient in accelerating tolerance in cultivars. Understanding about genomic loci governing traits responsible for tolerance and availability of molecular markers tightly linked with it is a prerequisite for marker assisted selection (Xu et al., 2012). A large amount of genomic data in the form of sequenced genomes and expression profiles are thus, impetuous for breeding for stress alleviation (Sonah et al., 2011; Tomar et al., 2014). Use of genomics-based technologies has made a great impact in crop improvement programs. Use of molecular markers in crop improvement for the accumulation of silicon (Si) in rice to enhance the tolerance of plant for abiotic stress is in vogue. Ma et al. (2004) used PCR-based markers for microsatellite (RM5303) and expressed sequence tag (EST, E60168) in mapping Si transporter gene during a bulk segregant experiments.

Besides crop breeding programs, a significant level of abiotic stress alleviation in plants can be achieved through the manifestations of plant-microbe interaction also. Omics approaches help to have deep insight into the mechanisms of established plant–microbe interactions (**Figure 2**). In a study of *Trichoderma-*plant interaction (*T. atroviride* and *T. harzianum* with tomato), Tucci et al. (2011) reported the impact of genotypic characteristics of plants for modulation of microbe–plant interaction leading to its effect on plant growth and stress alleviation. Growth promoting and stress alleviating activity of *T. atroviride* on tomato is demonstrated through degradation of IAA in the rhizosphere and ACC deaminase activity (Gravel et al., 2007). A putative sequence of ACC-deaminase found in *Trichoderma* genome was confirmed by gene silencing through RNAi (Viterbo et al., 2010; Kubicek et al., 2011). Expression of dicarboxylate transporter LjALMT4 responsible for carbohydrate translocation in plants was reported in *Lotus japonicas* genome by Takanashi et al. (2016). The gene silencing strategy of Hb1 gene to enhance NO production and up-regulation of CBF regulon could be a way to engineer crops in improving cold tolerance (Sehrawat et al., 2013). Kumari et al. (2015) reported IST to salinity in soybean by *Pseudomonas* sp. AK-1 and *Bacillus* sp. SJ-5 inoculation. Results indicated that superior tolerance to salt stress may be observed due to proline accumulation and lipoxygenase activity.

Koussevitzky et al. (2008) reported that Apx1, a gene coding for cytosolic ascorbate peroxidase 1 is specifically required for tolerance to drought and heat stress in *Arabidopsis.* Ectoine is a compatible osmolyte responsible for salt tolarance in *Halomonas elongata* OUT30018. Three genes for ectoine biosynthesis were cloned and transferred to tobacco plant (*Nicotiana tabacum L.*) cv Bright Yellow 2 (BY2) which caused increase in tolerance to hyperosmotic shock by accumulation of ectoine and exhibited normal growth under such conditions (Nakayama et al., 2000). Identifying the genes and their regulation helps breeders in generating better varieties for stress tolerance. Use of multi-omics strategies yield highly efficient and reliable outcome that are useful to facilitate methodical experiments (**Figure 2**). Chilling tolerance in *Miscanthus* grass is a desirable trait that often varies in different cultivars. Molecular expression of relevant genes for the accumulation of carbohydrates creates differentiation among varieties for chilling tolerance. The impairment of tolerance among varieties can be predicted by molecular marker of sensitive genes like TF MsCBF3 expressed in sensitive genotypes (Purdy et al., 2013).

Stress due to submergence affects more than 15 million hectares of rainfed lowland rice in different parts of Asia (Neeraja et al., 2007). Thirteen percent of the total land area of the world is affected by problems of flooding or anoxia (Cramer et al., 2011). In rice, submergence tolerance is governed by a single major quantitative trait locus (QTL) found on chromosome 9 (Toojinda et al., 2003). Neeraja et al. (2007) used molecular markers for *Sub1* gene in backcross breeding program with recurrent parant *Swarna*. This *Sub1* provides tolerance in sensitive mega varieties. *Sub1A* is now confirmed of being the primary contributor to submergence tolerance (Septiningsih et al., 2009). This QTL has provided a great opportunity for marker assisted backcrossing (MAB) for developing submergence tolerance in mega varieties.

Genomic analysis of both the host and associated microbial communities especially phyllosphere-associated microbial communities permits to access the system involved for the smooth functioning of associative interactions (**Figure 2**). Several studies have outlined the role of different genes from associated bacteria. Plants donate indispensible moleculr counterparts to facilitate and maintain the biological system involved at the associative interface. The genotypic diversity of plants has significant influence on the interactive process. The response of the roots of *SUNN1 Medicago truncatula* toward elevated levels of nitrate gets markedly affected with the advent of associative rhizobia because *SUNN1* exhibited no impact showing the response of *SUNN1* under limiting nitrogen environment in presence of associative rhizobia (Jin et al., 2012). There are evidences on the influence of *Nod* factors from associative microbes on the pattern of root development and smooth functioning of symbiotic association (Olah et al., 2005; Oldroyd, 2013). Unlike nodulating plants, widely cultivated cereals lack a system to acquire nitrogen with the help of nodulation. Some diazotrophic microbes manage to enter and colonize root tissues via mechanical injuries caused during root growth (Gaiero et al., 2013). However, knowledge of such interactions is scarce.

## Metagenomics

**FIGURE 3:**
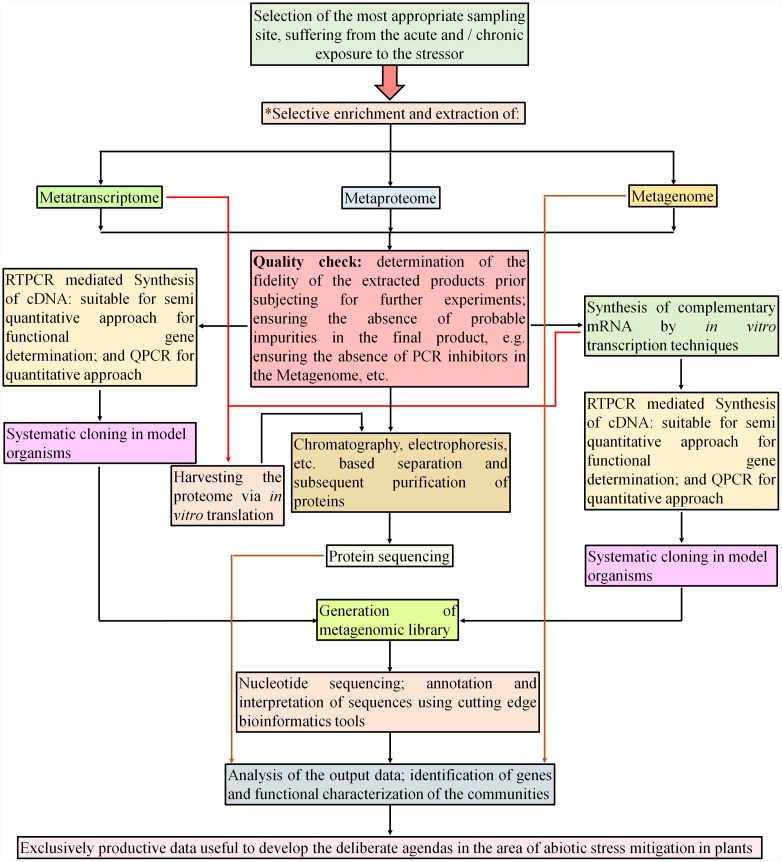
**Meta-omics approaches to exploit yet-unexplored environmental population of microbial communities that have major impact on plant roots and support plants against stresses.** Metatranscriptomics and metaproteomics are relatively new approaches to characterize functional attributes of microbial communities that have not yet been cultured. The approach could generate a deeper snapshot of major metabolic pathways and interactions and dominance of functional microbial communities in the rhizosphere of crop plants facing multiple environmental stresses. (^∗^Enrichment techniques in metagenomics. In order to trace out less abundant genes from the environment, these techniques are usually employed. In induced enrichment approach, the physico-chemical factors such as nutrients, temperature, acidity/alkalinity, xenobiotic compounds, etc. (Eyers et al., 2004; Bertrand et al., 2005) are used to enrich the respective populations *in situ*. These factors are either directly implemented in the microbial habitat itself or used in simulated *in situ* laboratory conditions. The natural sample enrichment is mainly dependent on executing fine criteria while proceeding for sampling of an environment. The naturally predominating bio-geo-physico-chemical situations need to be considered, as they are the key factors for selective natural enrichment of genes, e.g., sites contaminated with xenobiotic compounds and habitats with extreme environments can be expected to yield the genes participating in the metabolism of xenobiotic compounds and the genes participating in environmental stress tolerance respectively. The enrichment of nucleic acids from natural environment is principally carried out for the samples containing insufficient quantities of nucleic acids. It involves techniques such as affinity capture, differential expression analysis, stable isotope probing, e.g., addition of ^13^C labeled carbon source in the habitat. For the samples with low density of biomass, whole genome amplification technique is recommended to yield relatively larger quantity of nucleic acids (Abulencia et al., 2006; Binga et al., 2008). These approaches may work better with the samples collected from highly saline/sodic/drought affected, barren soils, where it is virtually difficult to cultivate the crop. The stress-genes of the little microbial community thriving in such harsh environments may provide novel guidelines for stress alleviation strategies in the crop).

The culture-independent approach for the analysis of microbial communities has been a powerful tool for resolution of yet-uncultured, unseen microbial diversity that plays various role in the plant rhizosphere (Chen and Pachter, 2005) (**Figure 3**). The approach referred to as metagenomics enables the user to acquire data related to the habitat-specific distribution of microbial communities with plant growth promoting (PGP), biocontrol, antibiotic producing and xenobiotic degrading traits. The approach helps to elevate likelihood of successfully directed attempts made to explore novel culturable flora from particular niches (Handelsman et al., 2007). High-throughput metagenomic sequencing is proving to be an extremely useful tool for improved understanding of PGP rhizobacterial communities (**Figure 3**). In a study on potato endophytes, two types of ACC-deaminase genes (*acdS*) homologous to that of *Pseudomonas fluorescens* for stress alleviation were found from PCR analysis. Analysis of clones present in metagenomic libraries helped in identifying entire *acdS* operon from uncultivated endophyte and revealed a transcriptional regulator gene *acdR* at upstream of *acdS*. This operon was found prominently in the genus *Burkholderia* (Nikolic et al., 2011). *Escherichia coli* clones from a pond water metagenomic library were studied to identify salt tolerance genes in uncultivable bacteria by growing at inhibitory NaCl concentrations of 750 mM. Genes from two clones encoding for proteins similar to a putative general stress protein (*GspM*) having GsiB domain with a putative enoyl-CoA hydratase (*EchM*) identified to have a role in salt tolerance. After purification, *EchM* was found to have crotonyl-CoA hydratase activity (Kapardar et al., 2010). These genes are of great utility in developing salt tolerant recombinant bacteria and also transgenic plants. Metagenomic study of an acid mine drainage 250 m belowground revealed the presence of mechanisms of adaptation to cold. Genes related with the survival at low temperature like anti-freeze protein, cold-shock proteins, compatible solutes production pathways and pH homeostasis were found in the metagenome of acid mine drainage (Liljeqvist et al., 2015). Such metagenomics data help to finding out new genes and mechanisms for cold stress alleviation. The role of bacterial endophytes that reside inside roots is largely unexplored because endophytic microbes which are cultured successfully represent only a fraction of the whole bacterial community that inhabit root interiors. Sessitsch et al. (2012) described endophytic bacterial residents of rice roots with the help of metagenomics approach. Metagenomic sequences obtained from endophytic cell extracts revealed that metabolic processes pertaining to the endophytic life style and functional features like quorum sensing and detoxification of ROS have their role in improving plant stress resistance (Sessitsch et al., 2012). Microbial communities have a fundamental impact on plant health and productivity. As a community, microbes interact with each other and with the host. This is a key phenomenon that increases resistance to diseases and stresses. To know the microbiome composition and describe its diversity and function, global approaches like metagenomics, metatranscriptomics and metaproteomics are being applied (**Figure 3**). Metagenomics also reveals functional potential of microbial communities in terms of the abundance of the genes involved in particular metabolic processes linked with stresses or stress alleviation mechanism. Similarly, metatranscriptomics can reveal kingdom-level changes in rhizosphere microbiome structure (Turner et al., 2013b) and metaproteomics can reflect community-wide gene expression, protein abundance and putative proteins that can be linked with functions after bioinformatics analysis (Turner et al., 2013b). Diversity profiling and colonization studies using metagenomics can also reveal quantitative colonization of a given host under the influence of stressor. This can yield valuable knowledge regarding stressor-induced alterations in the taxonomic and functional diversity of colonizing population if coupled with metatranscriptomic analysis (Turner et al., 2013b) (**Figure 3**). The coupled analysis thus achieved significantly help in understanding mitigation of stressor-influence over colonization process.

## Transcriptomics

Comparison of transcriptome profiles is helpful in identifying different sets of transcripts responsible for differences between two biologically different expressions in various conditions (Bräutigam and Gowik, 2010). Use of mRNA sequencing analysis and microarray technique to generate transcriptome level information is one of the important methodologies employed for studying plant-microbe interactions (Akpinar et al., 2015; Budak and Akpinar, 2015; Wang et al., 2016). Next-generation RNA sequencing study on *Sinorhizobium meliloti* revealed induction of genes for stress response in IAA overproducing strains (Defez et al., 2016). This study compared transcription profile of two *S. meliloti* strains, wild-type strain-1021 and an IAA overproducing derivative RD64. The genes coding for sigma factor RpoH1 and other stress responses were found to induce IAA overproducing strain of *S. meliloti*. Alavi et al. (2013) identified spermidine as a novel plant growth regulator during abiotic stress by transcriptome analysis of rapeseed and its symbiont *Stenotrophomonas rhizophila*.

Different miRNAs in rice, *Medicago, Phaseolus, Arabidopsis* and other plants have a regulatory role under abiotic stresses like drought, salinity and cold (Trindade et al., 2010). miRNAs are non-coding RNAs of 19–23 nucleotide length having regulatory role in several biological processes (Budak et al., 2015). Regulatory role of miR393 was found for salinity tolerance in *Arabidopsis* overexpressing osaMIR393 exhibit tolerant to salt excess (Gao et al., 2011). Zhao et al. (2009) reported miR169 alleviating salinity and drought stress in rice by modulating expression of a nuclear transcription factor YA (*NF-YA*). In tomato, plants overexpressing miR169c which controls expression of gene(s) involved in stomatal activity confer drought tolerance (Zhang et al., 2011). *Bvu-miR13* regulates WD-repeat proteins which plays crucial role in stress tolerance in cucumber (Li et al., 2014). Apart from regulating TFs, miRNAs also target stress signaling pathways which are responsible for root development, leaf morphogenesis and stress response (Curaba et al., 2014). Thirteen mature miRNAs were identified using *in silico* approach in *B. vulgaris* plants (Li et al., 2015). The activity of superoxide dismutases SOD1 and SOD2 mRNAs are targeted by miR398 that has a role in reducing ROS and secondary effects of abiotic stress (Kantar et al., 2011). Diverse classes of miRNAs alleviate stress by regulating differential cellular responses and metabolic processes like transcriptional regulation, auxin homeostasis, ion transport and apoptosis (Li et al., 2010). miRNA is also found to regulate aluminum stress response in plants (Lima et al., 2011). Comparison of miRNA expression in two different rice subspecies, *japonica* and *indica* differing in aluminum tolerance was done. RT-qPCR approach revealed 16 different kinds of responses of miRNA indicating a complex response under aluminum stress.

UV-B radiation and flooding (hypoxia) affects plants by inducing irreversible damage by generation of ROS (Blokhina and Fagerstedt, 2010). The up-regulation of SOD proteins and miR398 down-regulation is crucial under oxidative stress in *Arabidopsis* (Sunkar et al., 2006). Induction of miR398 and down-regulation of miR395 was observed in alleviating UV-B stress in *Populus tremula* (Jia et al., 2009). Low temperature severely affects sugar beet seedlings and sugar recovery from final harvest. Transcriptome profile of cold stressed plants was done by high throughput RNA sequencing from leaves and roots (Moliterni et al., 2015). Up-regulation of *CBF3* was reported from root tissues faster than the leaf tissues. Genes from *AP2/ERF* family that were known to participate in jasmonic acid mediated responses were also upregulated during cold stress (Licausi et al., 2013).

## Proteomics

Proteins play a crucial role in expressing plant stress responses since they directly reflect shaping of a phenotypic trait. Proteomic studies therefore, have become powerful tools for the exploration of physiological metabolism and protein–protein interactions in microbes and plants (**Figures 2** and **3**). The implications of proteomics is important for intra- and inter-microbial species and host–microbe interactions, where host-mediated signaling and tactic responses of related microorganisms are involved (Kosova et al., 2015). Such studies lead to generate a deeper understanding of the regulation of biological system by identifying several proteins as signal of changes in physiological status due to stress or factors responsible for stress alleviation (Silva-Sanchez et al., 2015). Therefore, a comparative analysis in stressed, non-stressed and microbe-associated plants can help to identify protein targets and networks. Proteomic studies for stress responses in crops have been studied extensively in plants including *Arabidopsis*, wheat (*Triticum aestivum*), durum wheat (*Triticum durum*), barley (*Hordeum vulgare)*, maize (*Zea maize*), rice (*Oryza sativa*), soybean (*Soybean max*), common bean (*Phaseolus vulgaris*), pea (*Pisum sativum*), oilseed rape (*Brassica napus*), potato (*Solanum tuberosum*) and tomato (*Lycopersicon esculentum*) (Liu et al., 2015; Kosova et al., 2015; Xu J. et al., 2015; Wang et al., 2016). Such studies reflected dynamic alternations in protein functional groups, proteins of signaling and regulatory pathways, TFs, protein metabolism, protein–protein interactions at interface, proteins and enzymes conferring several stress-related compounds, functions of structural proteins associated with the cell wall and cytoskeleton and identification of putative proteins using bioinformatics tools (Kosova et al., 2015).

Chen et al. (2015) assessed mechanisms of cold acclimation in alfalfa by proteomic analysis in cold tolerant (ZD7) and cold sensitive lines (W5). Cassava, a tropical crop sensitive to low temperature can modify its metabolism and growth to adapt to the cold stress. Proteomic study was carried out to understand the mechanism behind cold-tolerant process. Twenty differential proteins were found to have similar patterns in apical expanded leaves of cultivars SC8 and Col1046. Expression of proteome profile was found to link closely with changes in photosynthetic activity and peroxiredoxin expression levels. Principle component analysis reflected that electrolyte leakage (EL), chlorophyll content, and malondialdehyde (MDA) accumulation were the physiological indexes in determining cold tolerance in cassava (An et al., 2016).

A leucine-rich repeat receptor kinase (*Srlk*) was reported to function as an upstream regulator of salinity responsive genes in *Medicago truncatula.* It was found to be involved in sensing salinity stress and its response (de Lorenzo et al., 2009). This study revealed an interesting mechanism of sensing salinity stress. Based on proteome profile of barley at different water stress conditions, Ghabooli et al. (2013) proposed that *P. indica* mitigate drought stress by photosynthesis stimulation releasing energy and higher antioxidant production. Wang et al. (2016) screened a novel gene *Ds-26-16* from the cDNA library of 4M salt-stressed *Dunaliella salina*. This gene was found to confer salt tolerance in *E. coli. Haematococcus pluvialis* and tobacco. Proteomics data by iTRAQ studies reflected that *Ds-26-16* up-regulates TFs for stress responses like ROS alleviation, osmotic balance, and energy metabolism in *E. coli.*

The diversity of metabolic pathways existing amongst the microbes makes them more responsive toward stress conditions. It is important in the protocols implemented for the elucidation of plant-elaborated responses against stress. Unlike routine proteomics approaches which are more focused toward a single organism, the role of metaproteomics that deals with multiple metabolic interactions occurring simultaneously in an ecosystem is the need of the time (**Figure 3**). This could help to resolve better significance of interdependence between various microbial communities in an agro-ecosystem alongwith their interactions with the host plant. The protocols for protein extraction from the environmental samples are most important success-milestone in the area of metaproteomics (**Figure 3**). Recent advancements in protein sequencing are the key step for the identification of proteins from diverse species (Cordwell et al., 1995, 1997; Shevchenko et al., 2001). The complexity of metaproteome makes resolution and analysis quite difficult. However, recent approaches in extraction and analysis of successful environmental metaproteome could yield decisive output and establish a better correlation among the omics data and response mechanisms among organisms toward stresses (Schulze, 2005; Schweder et al., 2008).

Most of the environmental proteomic experiments are limited to model organisms cultured. They particularly highlight the exceptional ability of the organisms, e.g., tolerance to salinity, sodicity, temperature, low water availability, toxic metals and radiation etc. The proteomic studies of the organisms lead toward better understanding of fine mechanisms being executed by them. Moreover, the same also stands helpful for the confirmation of the probability of their exploitation for expected induction of the said metabolism in diverse environments. The laboratory experiments allow a better grasp of the protein profile in a controlled environment, however, it contrasts the fact that the expression profile varies with changes in environmental conditions.

The Haloarchea and Halobacteria are gaining strong attention in present era due to their ability to thrive in high salt environments. PGP ability of organisms can be implemented conveniently in saline and sodic soils for the alleviation of respective stresses encountered by the crops. This will prove beneficial for yield improvement in stress-prone areas. Harvesting and implementing metabolites that can confer halotolerance from microorganisms growing in the area of high salt stress with other combined stresses may find important applications in the crop improvement programs. Culturing of these organisms under *in situ* stress conditions in laboratory is the simplest approach to induce the production of effective metabolites that when applied on plants, could impart tolerance against stresses. Similarly, to cope with the most agonizing problem of xenobiotic compounds, the genus *Pseudomonas* is the best considered one, particularly because of its unique ability to degrade enormous amount of carbon sources, especially of xenobiotic nature. The hydrocarbon degradation by *Pseudomonas* is well known. The proteomic experiments for *Pseudomonas* have been designed principally to focus on the recalcitrant, xenobiotic compounds in addition to the toxic organic pollutants (Lupi et al., 1995; Reardon and Kim, 2002; Kim et al., 2007). Species of *Pseudomonas* have been well characterized for their PGP traits such as siderophore production (Ferret et al., 2014; Cunrath et al., 2015), secretion of plant growth stimulating substances (Pereira and Castro, 2014; Balcazar et al., 2015; Sorty et al., 2016), and biocontrol against phytopathogenic organisms (Chet and Inbar, 1994; Natsch et al., 1994; Acebo-Guerrero et al., 2015; Wang et al., 2015). Characteristic versatile metabolic scope and unique biofilm forming ability of the members of this genus (Kertesz et al., 1993; Sauer et al., 2002, 2004; Arevalo-Ferro et al., 2005; VerBerkmoes et al., 2006) permits these species to thrive well under diverse environmental conditions, thus making them most effective inoculants for field application.

The proteomic exploration of methylotrophic bacteria is also an active area of interest today. Methylotrophs constitute a major portion of phylosphere community, typically leaf surface, where one-carbon substrate, methanol is easily available via transpiration activity. Pink-pigmented facultative methylotrophic (PPFM) bacteria are predominant and explored largely for their ability to release plant-growth regulation molecules (Meena et al., 2012; Araujo et al., 2015; Dourado et al., 2015). Many studies have successfully demonstrated PGP potential of these organisms under various conditions (Tani et al., 2012; Yim et al., 2013). Detailed investigations about the proteomic insights of these characteristic phyllosphere-community members helped to get novel ideas regarding involvements of proteins in survival mechanism of organisms under relatively harsh environments, generally encountered on leaf surfaces, where in addition to intense radiation, there exists frequent scarcity of nutrients. Additionally, their potential to secrete plant-growth regulators may come to a large-scale execution. It is, therefore, needful to elucidate deep molecular insights of PGP microbial communities, chiefly involved in stress alleviation to acquire the data regarding mechanisms involved in such processes. The identification of proteins involved in these processes is sufficient to create a boom in stress alleviation strategies at the molecular level where direct implementation of active molecules were thought upon instead of employing the whole organism.

In plants, the study of protein expression of different lines is helpful in selecting cold-tolerant lines for crop improvement. It is evident from earlier studies that cold-tolerant lines showed 14 differential proteome expressions in cold acclimation of sunflower (Balbuena et al., 2011). Proteome analysis also reveals possible mechanisms for chilling mitigation in plants and cross tolerance mechanisms (Yuan et al., 2015; Meng et al., 2016). Once the database of responsive and blocked genotypes is made for particular abiotic stress, it can be used as a marker in differentiating stress responsive genotypes. Santos et al. (2016) has made GeLC–MS/MS based proteomic profiling for large-scale identification of proteins from *Araucaria angustifolia* embryogenic cell lines. In total, 106 proteins were differentially expressed between the responsive and blocked type lines for abiotic stress. Two proteins at early stage were identified to be related with blocked cell lines only. These proteins can be an indicative to blocked cell lines at early stage of plant development.

## Metabolomics

The scope of metabolomics involves characterization of all the metabolites elaborated by an organism under the influence of given environmental conditions. The metabolome of an organism directly correlates with diverse pathways being operated inside the cell which in turn reflects the availability of corresponding genetic information. The metabolome varies largely with alterations in surrounding environment that induce direct physiological alterations in an organism (Bundy et al., 2005). Similar situations of physiological state are expected in those organisms which are supposed to thrive well under the stress conditions. It is, therefore, important to acquire detailed knowledge of metabolome of an organism both in normal and under-stress physiological status, the subtraction of which will yield the presence/absence of typical signature metabolites of interest. This will be helpful in identifying alterations induced within the pathways and induction of typical stress-inducible genes (**Figure 2**). Metabolomics is increasingly being used for generating deep insights into abiotic stress responses (Jorge et al., 2015; Jia et al., 2016). Recent high throughput developments in the area of molecular detection techniques have given boost to metabolomics studies (Hollywood et al., 2006; Morrow, 2010). Studies highlight the presence of different bioactive chemicals (Burns et al., 2003; Ketchum et al., 2003) in plant metabolome. This observation correlates with the reports pertaining to the identification of various signal molecules secreted by plants to attract and induce important biochemical pathways in colonizing microbial population (Zhang and Cheng, 2006; Micallef et al., 2009).

*Trichoderma* spp. produce auxins which stimulate plant growth by alleviating stress (Contreras-Cornejo et al., 2009). Two secondary metabolites, harzianolide and 6-pentyl-a-pyrone of *Trichoderma* was reported to exhibit auxin-like effects in etiolated pea stem (Vinale et al., 2008) and enhance plant growth. Variations induced by changing environmental situations in plant metabolism also affect secretion pattern and nature of secreted molecules (Martínez-Cortés et al., 2014) thereby affecting the level of root colonization. Microbial molecular signaling mechanisms in the rhizosphere are also affected in a similar manner but this is yet to be explored.

Plants accumulate different metabolites like trehalose, glycine betain, IAA etc. in response to abiotic stresses. Allen et al. (2009) reported that mere accumulation of a specific compound does not indicate stress tolerance, but it is the adjustment of flux to different pathways of defense and growth which decides tolerance. Modulation of stoichiometry and metabolism is reported as mechanisms to maintain optimum fitness in plants (Rivas-Ubach et al., 2012). Time-series experiments with *Arabidopsis thaliana* indicated that metabolic activities respond more quickly than that of transcriptional activities to abiotic alterations (Caldana et al., 2011).

The conditions, available within surrounding environment influence pathways operating in the microbial cell, thereby affecting the metabolome. It is evident that the same must affect their overall performance in surrounding microenvironment and within the ecosystem to a greater extent (Tringe et al., 2005; Raes and Bork, 2008; Jiao et al., 2010) in terms of the interactions evident within and between the inhabitants therein. Microbial metabolic products have been involved in both direct as well as indirect plant growth promotion. It is well known that many of the rhizosphere bacteria show the ability to produce plant growth stimulating biomolecules like cytokinins, gibberelins, etc. (Williams and de Mallorca, 1982; Robin et al., 2006). Variety of microbial metabolites including IAA, gibberelins, siderophores serve the purpose. Recently the IAA produced by *Pseudomonas* sp., *Rhizobium* sp., *Enterobacter* sp., *Pantoea* sp., *Marinobacterium* sp., *Acinetobacter* sp., and *Sinorhizobium* sp., has been shown to influence the germination and seedling growth in wheat under saline conditions (Sorty et al., 2016). Similarly, the strains of *Bacillus* sp. having phosphate solubilizing potential successfully improved the yield and quality of fennel in semiarid saline soil (Mishra et al., 2016). The solubilization of phosphate is mainly attributed to the low molecular weight organic acids produced by the microbes. Microbial siderophores also play an important role toward the biological availability of iron to plant roots, for instance, the siderophores produced by *Pseudomonas fluorescens* C7 successfully supplemented the iron to *Arabidopsis thaliana* (Vansuyt et al., 2007). Although the siderophore production by the microbes seems influenced by biogeochemical factors, they also help in the alleviation of the stress imposed by heavy metals (Diels et al., 2002). Many microbes show high degree of environmental dependency for optimal siderophore production. Sorty and Shaikh (2015) reported reduced iron uptake by both sediment as well as soil magnetotactic bacteria under acidic conditions and the probable cause was attributed to the conversion of Fe^+++^ to Fe^++^ under acidic conditions that could have interfered with the siderophore mediated iron uptake system. This signifies the need of keen evaluation of *in situ* mechanisms influencing microbial metabolism. Moreover, the majority of these metabolites are yet to be identified. The cutting edge metabolomics technology could serve as a powerful tool for the evaluation of these metabolites and environmental interventions in the microbial metabolism *in situ*. Many microbes from the ecosystem show interdependence with respect to the substrate utilization and metabolite exchange forming the basis of succession. Moreover, the same is applicable in the area of biodegradation of recalcitrant as well as xenobiotic compounds too, where co-metabolism is shown to play the principle role. This involves simultaneous oxidation of non-substrate compounds with that of true substrates during vigorous growth of bacteria. The members belonging to the genera *Pseudomonas, Flavobacterium, Bacillus, Azotobacter, Microbacterium, Hydrogenomonas, Achromobacter* and *Xanthomonas* are predominant co-metabolisers in the ecosystem (Beam and Perry, 1973). This property of co-metabolism has significant implications in the studies depicting biochemical pathways, particularly involved in the metabolism of polycyclic and polyaromatic compounds (Horvath, 1972; Chauhan et al., 2008). Metabolomics studies of these processes provide the information on the enzymes involved in the conversion and pathways they participate in, thereby raising the probability of their large-scale exploitation to the sites where the native ecosystems have encountered the stress conditions due to the accumulation and/or contamination of xenobiotic and recalcitrant compounds. Many hydrocarbons such as *p*-isopropyltoulene, *n*-butylcyclohexane, *n*-butylbenzene, ortho and para xylene etc. are actively co-metabolized by the members of genus Nocardia (Davis and Raymond, 1961; Raymond et al., 1967). The inimitable metabolic power of such organisms highlight strong implementable ability of PGP members of such genera to remediate stresses imposed by contaminated soils on crop and thus pave the way for bioremediation.

The quantitative metabolomics studies also permit measurements of cellular processes with high accuracy and precision (Noack and Wiechert, 2014). The high-throughput mass spectrometric profiling of cellular metabolites of plant-associated microbes under the influence of stressors could reveal the level of interference by the stressor in the overall cellular homeostasis (**Figure 2**). The communication between plants and soil microbial community represents a bilateral process involving root exudates and microbial-elaborated signal response molecules (Oldroyd and Downie, 2008; Inceoglu et al., 2011; Peiffer et al., 2013). The augmentation of rhizosphere with exogenous microbial metabolites also needs prior insights into the microbial metabolism. This includes the ratio of cellular abundance, biomolecules elaborated under normal and optimal circumstances, quantitative leakage, participation of plant signals in the cascade and resulting counter response of microbes. It could be thoughtful to enrich such biomolecules in the rhizosphere that are down-regulated due to the influence of the stressor. Similar is applicable to the probable management of stressor-responsive biomolecules influencing overall communication process between the host and microbe. The altered plant root exudation under the influence of stressor fails to induce cascades described above in microbial systems that transpire otherwise.

The accessibility of nucleotide data has been one of the value additions for metabolomics studies (NCBI). This ensures smoothening of future experiments targeting systems biological perspectives. The recruitment of microbial-originated biomolecules and/or *in vitro* synthesized metabolites under simulated conditions in the phyllosphere have been demonstrated recently (Sorty et al., 2016). This mainly deals with the fact that live microbes, under stressed environment fail to express vital genes for PGP activity. However, the impact of enrichment of rhizosphere with the appropriate quantity of such metabolites needs thorough evaluation. The insights to host metabolomics are also beneficial to acquire knowledge regarding the influence of host on post-colonization metabolism of microbes. This could open the gateway for simulation of highly complex endophytic environment.

Rhizosphere community also represents multifaceted system involving biogeochemical cycling and exchange of nutrients, leaving an excellent platform for gaining deep insights in to the systems microbiology. Enormous pathways are simultaneously operated by diverse members of microbial community. Environmental factors have maximum influence on the smooth operation of such pathways. Arrival of stressor/altered environmental situation ultimately diverts overall functionality of microbial system, thereby inducing variation in the community structure. The understanding of biochemical links within and between the members of an ecosystem is necessary to acquire the phenotype-level knowledge in a given biogeochemical state of event (Breitling et al., 2008).

## Conclusion

Plant responses toward various abiotic stresses and microbe-mediated stress mitigation strategies in plants have been studied on sound grounds of molecular, biochemical, physiological and ultrastructural parameters. Such studies have been carried out encompassing different omics approaches (genomics, metagenomics, metatranscriptomics, proteomics, metaproteomics and metabolomics) that strengthened our understanding behind the mechanisms of microbial interactions, gene cascades and metabolic pathways, accumulation and enhancement of various metabolites, proteins, enzymes and up- and down regulation of different genes. Such studies could yield dynamic data related to combined responses of plants to multiple stresses, and the same is also pertinent with the naturally associated or artificially inoculated microorganisms. These studies provide new directions for improvising the existing protocols in the field of plant–microbe interactions under stress, and use of microorganisms and microbial metabolite molecules for alleviation of diverse stresses encountered by plants. The expected outcomes are facilitating germination, superior sustenance, enhanced ability to combat adverse conditions of environment and superior yield in plants because of the use of microbe-elaborated molecules. Due to limitations regarding the sustenance of microbes in diversity of stress environments and variable responses at phenotypic level, it is always suitable to implement microbe-derived natural products that are capable of performing expected job of stress mitigation irrespective of the environmental situations. To conclude, we strongly advocate that there is a need to put greater attention on in-depth studies pertaining to identification, trait characterization, compatibility assessment, delivery methods and impact of application of microbes isolated from diverse environmental conditions for the mitigation of abiotic stresses in crop plants. We need to find out new roles for microbial metabolites that are being produced under stressed environmental conditions. Established evidences exist to support the role of microbe-mediated plant interactions in stress mitigation under diverse climatic and edaphic conditions. However, more focused omics-based research data generation following integrated approaches encompassing genomics, metagenomics, proteomics and metabolomics studies on specific plant–microbe-abiotic stress system will be needed to resolve many facts behind precise mechanisms of stress tolerance/mitigation in the crop plants.

## Author Contributions

KM proposed concept, KM and AS collected data and wrote the manuscript, KC and UB collected data, AP and PG added abiotic stress in plants, DS, RP, PS, HS, KK, VG, and PM contributed for omics data and edited the manuscript.

## Conflict of Interest Statement

The authors declare that the research was conducted in the absence of any commercial or financial relationships that could be construed as a potential conflict of interest.

## Footnotes

1 http://www.fao.org/docrep/010/a1075e/a1075e00.htm

2 http://www.fao.org/docrep/meeting/024/md324e.pdf
